# The role of long non-coding RNA H19 in infertility

**DOI:** 10.1038/s41420-023-01567-y

**Published:** 2023-07-28

**Authors:** Yuanyuan Peng, Renhao Guo, Bei Shi, Da Li

**Affiliations:** 1 Center of Reproductive Medicine, Shengjing Hospital of China Medical University, Shenyang, 110004 China; 2 NHC Key Laboratory of Advanced Reproductive Medicine and Fertility (China Medical University), National Health Commission, Shenyang, 110004 China; 3 Department of Physiology, School of Life Sciences, China Medical University, Shenyang, 110122 China

**Keywords:** Infertility, Predictive markers

## Abstract

Infertility is defined as the failure to conceive after at least one year of unprotected intercourse. Long non-coding RNAs (lncRNAs) are transcripts that contain more than 200 nucleotides but do not convert into proteins. LncRNAs, particularly lncRNA H19, have been linked to the emergence and progression of various diseases. This review focuses on the role of H19 in infertility caused by polycystic ovary syndrome, endometriosis, uterine fibroids, diminished ovarian reserve, male factor, and assisted reproductive technology-related pathology, highlighting the potential of H19 as a molecular target for the future treatment of infertility.

## Facts

1. LncRNAs play an essential role in the modulation of fertility.
2. LncRNA H19 has been linked to endometriosis, diminished ovarian reserve, polycystic ovary syndrome, uterine fibroids, and male factor infertility.
3. The clinical implications of lncRNA H19 in the treatment of reproductive endocrine diseases needs to be a focus of future research.

## Open questions

1. What are the characteristics of lncRNA H19-related infertility?
2. What are the etiological molecular mechanisms that involve lncRNA H19 in different causes of infertility?
3. What is the relationship between lncRNA H19 and assisted reproductive technology-related infertility?

## Introduction

Infertility is defined as the absence of pregnancy after at least one year of unprotected intercourse. It has become a global health problem that affects 15% of reproductive-age couples [1]. Infertility can occur in both males and females. While approximately 85% of infertility cases are caused by ovulation abnormalities, male-factor infertility, and tubal illness, unexplained infertility affects remaining 15% of infertile couples [2].

Epigenetics has provided recent and new insights into infertility. Epigenetic modifications, characterized by histone modifications, DNA methylation, chromatin remodeling, and RNA-based mechanisms, play crucial roles in many biological processes, including spermatogenesis and ovulation [3–5]. An increasing volume of research illustrates that long non-coding RNAs (lncRNAs), including lncRNA H19, play an essential role in the modulation of fertility [6]. H19 has been linked to several reproduction-related diseases, such as endometriosis [7], diminished ovarian reserve (DOR) [8], polycystic ovary syndrome (PCOS) [9], uterine fibroids (UFs) [10], and male factor infertility [11]. This review highlights the specific effects of lncRNA H19 on both female and male infertility, summarizes the role of H19 in infertility, and provides a theoretical foundation for its potential use as a novel molecular target for infertility treatment.

## Structure of the H19 gene locus

**Fig. 1:**
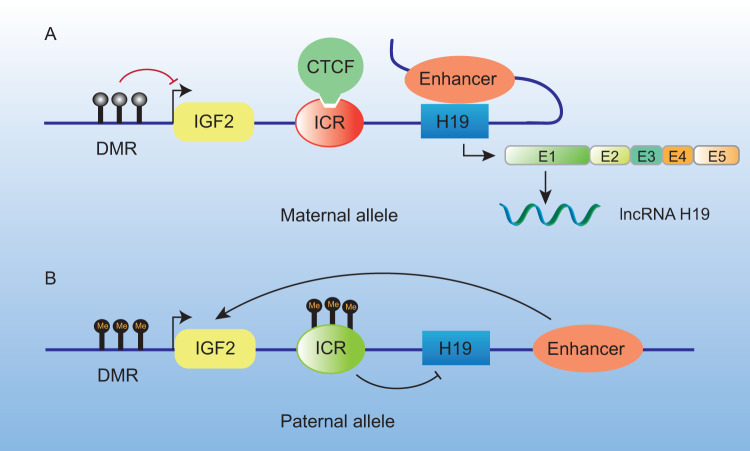
H19 expression is controlled by the ICR on the IGF2-H19 locus. **A** The maternal allele produces H19. The downstream enhancer cannot interact with the IGF2 promoter region because CTCF is bound to the unmethylated ICR, but it can interact with the H19 promoter. **B** The ICR is hypermethylated, preventing CTCF from binding and enabling interaction between the enhancer and the IGF2 promoter region, which suppresses the production of H19. CTCF CCCTC binding factor; DMR differentially methylated region; E1-5 Exon1-5; ICR imprinting control region, IGF2 insulin-like growth factor 2, lncRNA long non-coding RNA.

The H19 gene belongs to a conserved gene cluster on human chromosome 11p15.5 and on mouse chromosome 7 where insulin-like growth factor 2 (IGF2) also resides [12]. The gene contains four small introns and five exons. Following transcription, H19 is capped, spliced, polyadenylated, and exported to the cytoplasm [13]. The imprinting control region (ICR) for the IGF2 and H19 genes is situated 2.5 kb upstream of the H19 promoter region. IGF2 and H19 are expressed by the paternal and maternal alleles, respectively, and are both regulated by the H19-differentially methylated region (DMR) [14]. The zinc-finger protein CCCTC binding factor (CTCF), which binds to the unmethylated maternal ICR and inhibits IGF2 transcription via a downstream enhancer, controls the reciprocal expression of IGF2 and H19 in the same tissues [15] (Fig. 1). Under normal circumstances, the ICR is hypomethylated at the maternal allele and hypermethylated at the paternal allele; this differential methylation controls the normal expression of IGF2 and H19 [16]. H19 is highly conserved and produced in the skeletal muscle, heart, uterus, mammary glands, and ovaries throughout the early phase of embryogenesis and in adult tissues [17].

## H19 and polycystic ovary syndrome

PCOS is the major reason for anovulatory infertility [18]. It is distinguished by a series of interrelated reproductive and metabolic abnormalities, including gonadotropin secretion disorders, increased androgen secretion, chronic anovulation and polycystic ovary morphology, insulin resistance (IR), and obesity [19]. Downregulation of H19 affects PCOS, including amelioration of hyperandrogenemia [20], inhibition of matrix metalloproteinases (MMPs) associated with ovarian remodeling [21, 22], and inhibition of granulosa cell proliferation [23].

Some studies have reported that H19 expression is highly upregulated in the peripheral blood leukocytes of patients with PCOS compared with those of normal controls [9]. Women with PCOS frequently use metformin, a biguanide that reduces blood glucose levels in patients with hyperglycemia and type 2 diabetes [24]. Chen et al. demonstrated that H19 is a key target of metformin in PCOS treatment [21]. Remodeling of the intrafollicular microenvironment and follicular development requires the coordinated action of proteases and protease inhibitors [22]. Serum levels of MMP-9 and MMP-2 are elevated in patients with PCOS [25]. It was found that reduced levels of MMP-2 and MMP-9 in PCOS patients can lead to normal ovulation. Metformin reduced H19 expression by promoting methylation of the H19 promoter and inhibited expressions of MMP-9 and MMP-2 via the H19/microRNA (miR)-29b-3p pathway, thereby alleviating PCOS severity [21]. However, other studies have reported reduced H19 expression in a rat model of PCOS and that levels of H19 can be restored by co-treatment with sitagliptin and metformin. It was found that the co-treatment inhibited the phosphoinositide-3 kinase/protein kinase B signaling pathway, which in turn inhibited DNA methyltransferase 1 phosphorylation and nuclear translocation, leading to inhibition of H19 methylation and up-regulation of H19 expression. Outcomes included improved reproductive hormone homeostasis and reduced ovarian polycystic changes and IR [26].

In addition, researchers found that H19 plays a role in hyperandrogenemia in PCOS by regulating steroid 17alpha-monooxygenase (Cyp17). Deletion of H19 in mouse models resulted in decreased Cyp17 levels in the ovaries and decreased serum testosterone [20]. Increased testosterone synthesis in PCOS is partly due to the higher activity of cytochrome P450, family 17, subfamily A, polypeptide 1 (Cyp17A1), a rate-limiting enzyme of androgen production in the gonads and adrenal cortex [27]. In addition, patients with PCOS had higher H19 levels of ovarian and circulation compared to the control group. These findings imply that H19 deficiency may inhibit androgen synthesis via the Cyp17-mediated pathways. Conversely, excess H19 may have an impact on the development of PCOS-related hyperandrogenemia [20].

Moreover, H19 targets miR-19b to modulate the expression of connective tissue growth factor (CTGF), thereby promoting ovarian granulosa cell proliferation [23]. Increased expression of H19 has been detected in ovarian tissues and granulosa cells of patients with PCOS. Small interfering RNA-mediated knockdown of H19 in human granulosa-like tumor cell line (KGN) cells induces apoptosis and inhibits cell proliferation [28]. It has been suggested that CTGF contributes to granulosa cell proliferation and ovarian fibrosis. H19 promotes CTGF expression by competitively binding to miR-19b, which affects KGN cells proliferation [23].

In an association analysis between PCOS risk and H19 imprinted gene polymorphisms in an Iranian population, it was found that PCOS may be associated with imprinted gene polymorphisms, and that the rs2067051G >A allele is associated with a higher incidence of PCOS (odd ratio (OR) = 2.00, 95% confidence interval (CI) = 1.38–2.91, *P* < 0.01). The AG and AA genotypes increased the incidence of PCOS by 3.64 and 5 times, respectively (95% CI = 2.02–6.54, *P* < 0.01; 95% CI = 1.51–16.52, *P* < 0.01) [29].

**Fig. 2:**
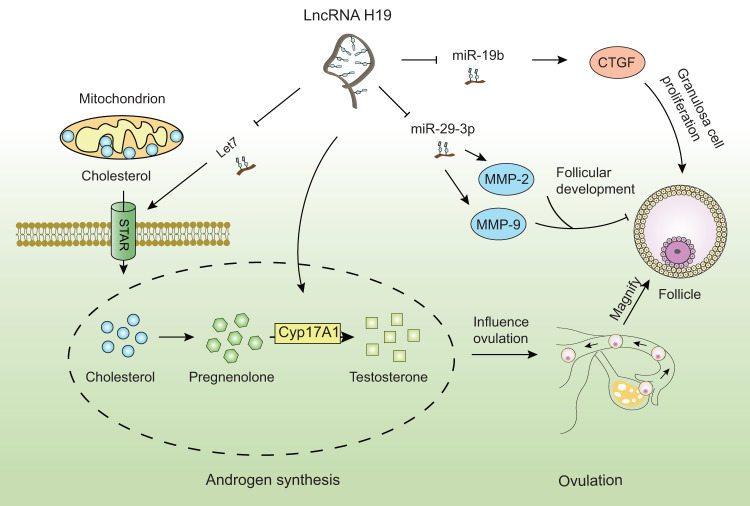
The expression and function of H19 in anovulatory diseases. H19 regulates androgen synthesis via Cyp17A1 pathways in PCOS. H19 affects granulosa cell proliferation via miR-19b/CTGF pathways in PCOS. H19 modulates follicular development via miR-29-3p/MMP-2 and MMP-9 pathways in PCOS. LncRNA H19 regulates ovarian function via Let7/STAR. CTGF connective tissue growth factor, Cyp17A1 cytochrome P450, family 17, subfamily A, polypeptide 1, lncRNA long non-coding RNA, miR- microRNA, MMP matrix metalloproteinase, PCOS polycystic ovary syndrome, STAR steroidogenic acute regulatory protein.

In combination, the evidence suggests that H19 could be a valuable biomarker for the detection of early-stage endocrine and metabolic abnormalities in PCOS (Fig. 2, Table 1).

**Table 1:** The expression and function of H19 in infertility-related diseases.

Levels of H19		Targets	Functions	References
Up	HGL5 cells isolated from the ovary of PCOS rats	miR-29b-3p	Regulated MMP-9/MMP-2	[21]
/	Human ovarian granulosa-like tumor cell line	miR-19b	Regulated granulosa cell proliferation and ovarian fibrosis	[23]
Up	Serum and cumulus cells of PCOS women	Cyp17	Interfered with androgen production	[20]
Down	PCOS model cells of rats	/	Regulated apoptosis of granulosa cells, reproductive hormone disorders, ovarian polycystic changs and insulin resistance	[26]
Up	Peripheral blood leukocytes of PCOS women	/	Risk factor for PCOS	[9]
Down	Serum of women with DOR	/	Correlated with AMH	[8]
Up	ESCs of women with endometriosis	miR-216a-5p	Promoted invasion and migration of ectopic ESCs	[32]
Up	ESCs of women with endometriosis	miR-124-3p	Inhibited the proliferation and invasion of ectopic ESCs	[33]
Down	Mononuclear cells from peritoneal fluid of patients with endometriosis	miR-342-3p	Inhibited Th17 cell differentiation and ESCs proliferation	[35]
Down	ESCs of women with endometriosis	Let7	Promoted proliferation of ESCs	[34]
Up	Human fibroids	Let7	Promoted proliferation of leiomyoma cells	[10]
/	H19 KO mouse	Let7	Limited the number of follicles that mature, produced estradiol, and ovulated	[48]
/	H19 KO mouse and human ovarian granulosa-like tumor cell line	Let7	Regulated STAR expression and progesterone production	[17]

*AMH* anti-Müllerian hormone, *Cyp17* cytochrome P450, family 17, *DOR* diminished ovarian reserve, *ESCs* endometrial stromal cells, *miR-* *microRNA*, *MMP-2* matrix metalloproteinase-2, *MMP-9* matrix metalloproteinase-9, *PCOS* polycystic ovary syndrome, *STAR* steroidogenic acute regulatory protein, *Th17* T helper cell 17.

## H19 and endometriosis

Endometriosis is an estrogen-dependent disorder that occurs during the reproductive period, leading to pain and infertility. It is distinguished by the growth of the endometrial lining outside the uterine cavity. None of the currently proposed pathogenic explanations (retrograde menstruation, coelomogenesis, or Müllerian residue) can account for the various endometriosis types. According to the most established model of endometriosis, the retrograde menstrual hypothesis, endometrial residue migrates through the fallopian tubes to the pelvis, where it implants in the peritoneum and abdominal organs, multiplies, and creates adhesions, producing persistent inflammation [30]. The menstrual cycle affects endometrial H19 levels. Endometrial stromal cells (ESCs) express H19 at a low level during the proliferative phase, this declines after ovulation, remains low during the early secretion phase, increases quickly on the 21st day of the menstrual cycle, and remains high until menstruation [7].

Studies have shown that H19 expression is noticeably higher in the ectopic endometrium of patients with endometriosis than in the normal endometrium [31]. Alterations in the H19/miR-216a-5p/actin alpha 2 (ACTA2) pathway may be involved in H19-mediated invasion and migration of ectopic ESCs, which could be an underlying cause of fibrogenesis or fibrosis in patients with endometriosis [32]. Furthermore, downregulation of H19 suppresses the proliferation and invasion of ectopic endometrial cells by regulating miR-124-3p and integrin beta 3 (ITGβ3) [33]. However, Ghazal et al. proposed that the expression of H19 in the ectopic endometrium of patients with endometriosis was considerably lower than that in normal controls. Reduced H19 activity increases Let7 activity, which suppresses IGF1R expression at the post-transcriptional level, and lowers ESC proliferation, this may contribute to reduced implantation receptivity, and it is linked to infertility [34].

The overexpression of H19 reduces T helper cell 17 (Th17) cell differentiation and ESC proliferation by modulating miR-342-3p/immediate early response 3 (IER3). Additionally, H19 overexpression inhibits the growth of endometrioid-like lesions generated by Th17 cell differentiation in vivo [35]. The significance of H19 overexpression for prognosis has also been assessed using The Cancer Genome Atlas database, which revealed that patients with endometrial/cervical cancer with H19 overexpression exhibited a reduced non-recurrence survival rate (hazard ratio (HR) = 2.261, *P* < 0.05) and a reduced overall survival rate (HR = 2.710, *P* < 0.05). Moreover, a multivariate Cox regression analysis revealed that H19 overexpression can be considered an independent predictor of an adverse outcome (HR = 4.099, *P* < 0.05) [36].

In other studies, endometriosis was prevented in naked mice by downregulating the H19 gene [37]. Infertility, bilateral ovarian lesions, recurrence, increased CA125 levels, and revised American Fertility Society stage were all related with expression of H19 in the ectopic endometrium [7]. Infertile patients displayed more evident H19 overexpression in both ectopic and eutopic endometria, implying that H19 may play a role in the incidence and progression of infertility in endometriosis [7]. H19 is a new prospective predictor of endometriosis recurrence and may be involved in the pathophysiology of endometriosis by influencing the proliferation and invasion of ectopic endometrial cells, particularly in the recurrence mechanism [7].

## H19 and uterine fibroids

**Fig. 3:**
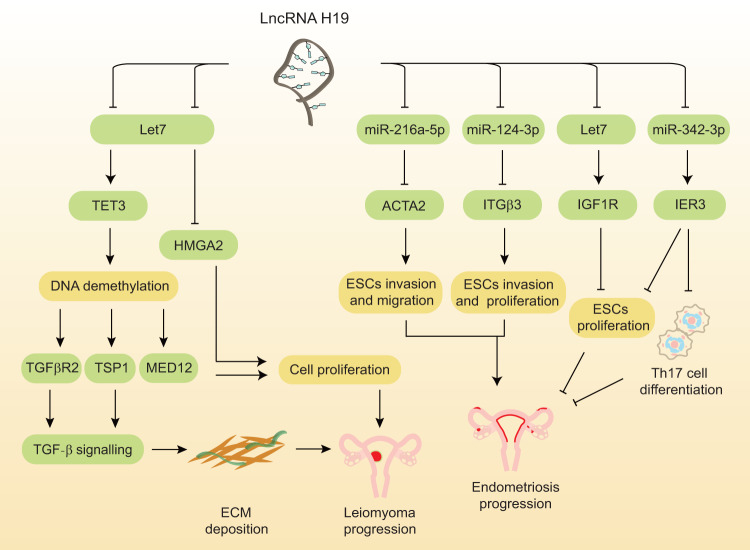
The expression and function of H19 in UFs and endometriosis. H19 regulates ECM deposition via Let7/TET3/TGFβR2 and TSP1/TGF-β pathways in UFs. H19 regulates cell proliferation via Let7/HMGA2 and Let7/TET3/MED2 pathways in UFs. H19 regulates cell proliferation via Let7/IGF1R in endometriosis. H19 modulates cell proliferation via miR-124-3p/ITGβ3 in endometriosis. H19 modulates cell proliferation via miR-216a-59/ACTA2 in endometriosis. H19 affects cell proliferation via miR-342-3p/IER3 in endometriosis. ACTA2 actin alpha 2, ECM extracellular matrix, ESCs endometrial stromal cells, HMGA2 high mobility group AT-hook 2, IER3 immediate early response 3, IGF1R insulin-like growth factor 1 receptor; ITGβ3 integrin beta 3, lncRNA long non-coding RNA, miR- microRNA, MED12 mediator complex subunit 12, TET3 Ten eleven translocation 3, Th17 T helper cell 17, TGF-β transforming growth factor-beta, TGFβR2 transforming growth factor beta-receptor 2, TSP1 thrombospondin-1, UFs uterine fibroids.

UFs are the most prevalent female tumors, with a higher prevalence found in infertile patients [38]. The deposition of large amounts of extracellular matrix (ECM) is the most conspicuous feature of UFs. Excessive ECM build-up contributes considerably to fibroid tumor development and rigidity. Growth hormones and soluble profibrotic substances that stimulate tumor growth are stored in the ECM. Thus, ECM could be a therapeutic target for UFs [39]. Transforming growth factor beta-receptor 2 (TGFβR2) and thrombospondin-1 (TSP1) are linked to aberrant ECM remodeling, and mediator complex subunit 12 (MED12) and high mobility group AT-hook 2 (HMGA2) have been linked to smooth muscle hyperplasia [40–43]. According to our previous findings, H19 expression was shown to be much higher in UFs than in the normal myometrium; H19 stimulated leiomyoma cell proliferation as well as the expression of MED12, HMGA2, ten-eleven translocation 3 (TET3), and ECM remodeling genes (Fig. 3, Table 1). H19 modulates gene expression via post-transcriptional and TET3-dependent epigenetic processes. The abnormally high expression of H19 in fibroids may influence the expression of fibroid-promoting genes and stimulate the proliferation of leiomyoma cells, which could be the mechanism underlying H19 involvement in UFs [10]. A study on the postoperative recurrence of UFs showed that the levels of H19 expression in patients with UFs after surgical treatment were significantly higher than those in healthy people, while the expression level of TET1 was significantly lower. Both H19 and TET1 are independent risk factors for the recurrence of UFs. In addition, they are quite effective at diagnosing and predicting the post-surgical recurrence of UFs [44]. Combined with the idea that single nucleotide polymorphisms (SNPs) of H19 are related to a growing risk of leiomyosarcoma and tumor size [45, 46], these findings indicate an essential role for H19 in the pathogenesis of UFs.

## H19 and diminished ovarian reserve

The number of follicles and oocytes (the ovarian reserves) decreases with age [47], resulting in decreased female fecundity and infertility. The levels of circulating and ovarian H19 are lower in women with DOR. A low oocyte yield upon retrieval is predicted by anti-Müllerian hormone (AMH), which is used as an important indicator to assess ovarian reserve. Studies have shown that women with low serum AMH levels also have low serum H19 levels [8]. H19 knockout (H19KO) mice exhibit traits that are comparable with those of AMH null (AMHKO) mice, such as rapid follicular recruitment and subfertility. H19KO mice have lower fertility and a faster follicular recruitment rate as well as more spontaneous growth of secondary, preantral, and antral follicles. H19KO animals display lower levels of ovarian AMH mRNA and proteins. AMH mRNA contains a functional Let7 binding site, suggesting that H19/Let7 regulation of AMH is mediated by non-coding RNA (ncRNA). Finally, superovulation in the absence of H19 leads to more estradiol and oocytes, implying that H19 limits the number of mature, estradiol-producing, ovulating follicles. Thus, the inhibition of AMH is at least partially mediated by H19, possibly via Let7, which marks this pair of ncRNAs as an important regulator of follicular cisterna establishment and maintenance [48]. These results indicate that H19 may be implicated in the regulation of ovarian reserves via the modulation of AMH. Additionally, H19 may also be valuable as an innovative diagnostic biomarker for ovarian reserve and premature ovarian insufficiency and has the potential to advance the diagnosis of DOR, particularly in circumstances when existing ovarian reserve testing is insufficient. Moreover, H19 may be of great significance in regulating steroid hormone production. The rate-limiting step of steroidogenesis is controlled by the steroidogenic acute regulatory protein (STAR); the deletion of H19 disrupts ovarian STAR in a H19KO mouse model (Fig. 2, Table 1), as well as abnormal progesterone production [17].

## H19 and male factor infertility

Male infertility is a multifactorial polygenic pathological disorder that affects approximately 7% of all males [49]. There are three causal factors of male infertility: genetic, developmental, and lifestyle factors [50]. Studies have shown that the methylation level of the H19 ICR in men with oligozoospermia and asthenospermia is significantly lower than that in healthy men, suggesting a connection between the hypomethylation of the H19 ICR and male infertility [11]. Furthermore, there are differences in DNA methylation of the H19 DMR between men with obstructive azoospermia and those with proven fertility and between men with reversed vasectomy and those with proven fertility [51]. Potentially, obstruction could be linked to imprinted genes. In testicular sperm, methylation at the H19 DMR is especially susceptible to modification [51]. Interestingly, the genotype of an SNP in the H19 DMR affected nearby DNA methylation levels [52]. Epimutations in the H19 DMR have been detected in 20% of men with oligozoospermia, and paternally expressed gene 1/mesoderm-specific transcript DMR epimutations have been found in 3% of men with the condition [53]. Moreover, studies suggested that methylation of the sixth CTCF binding site found in the H19 DMR differed significantly between high- and low-fertility bulls [54]. Another study concluded that 16 of 22 patients with oligo-astheno-teratozoospermia exhibited severe loss of the sixth CTCF methylation, which was closely related to sperm concentration [55]. The methylation profiles of CpG sites within the ICR of imprinted genes H19 and small nuclear ribonucleoprotein polypeptide N could be used as epigenetic biomarkers to evaluate male infertility with multiple sperm defects [56]. These studies suggest that the total methylation rate of patients with male infertility is low, and the aberrant methylation of the imprinting gene H19 is related to male infertility [57], indicating that H19 can be used as a biomarker to detect defects in human spermatogenesis.

## H19 and assisted reproductive technology-related pathology

Assisted reproductive technologies (ARTs) give infertile couples the chance to have children, but they also increase the chances of inheriting genetic and epigenetic changes [58]. Recent research has shown that the mouse reproductive tract contains a high number of lncRNAs involved in testicular development, spermatogenesis, sex determination, oocytic and embryonic development, trophoblast cell migration and invasion, and other reproductive processes. To implant an embryo, a receptive uterus must first be established. H19 mRNA was shown to be significantly expressed in the uteri of pregnant mice during the implantation window, with a peak on the fifth day of implantation [59]. It has been found that the expression of the endometrial imprinting gene H19 is downregulated in patients with repeated implantation failure after in vitro fertilization and embryo transfer or frozen embryo transfer [60]. Implantation failure is often caused by abnormal endometrial receptivity. ITGβ3, an microRNA Let7 target gene antagonized by lncRNA H19, is recognized as crucial for endometrial receptivity. The H19/Let7/ITGβ3 axis influences trophoblastic spheroid adherence to ESCs [61]. In rodent studies, ARTs have been linked to the disruption of genomic imprinting in embryos at the blastocyst stage. Between the eight-cell and morula stages, ART caused the loss of H19 ICR methylation, and the transfer of cleaved embryos to the uterus attenuated the deletion of H19 methylation caused by ART in mice [62].

Furthermore, H19 is the most studied allele in animal experiments involving processes such as ovulation induction and embryonic culture, and it has been investigated as a potential validation marker for epigenetic alterations in response to in vitro fertilization (IVF)/intracytoplasmic sperm injection [63]. One study provided new insights into the development of improved IVF procedures and the lifelong health of offspring by demonstrating that hypermethylation of the imprinted regulatory region of the H19-imprinted maternally expressed transcript is linked to IVF-induced placental dysplasia [64]. Considering that these abnormal methylation modifications may evade the demethylation process of the fertilized ovum and thus be passed down to future generations, resulting in illnesses, special precautions should be taken regarding potential epigenetic hazards during reproduction, particularly when utilizing ARTs.

## H19 and hyperprolactinemia

Hyperprolactinemia is an important cause of female infertility [65]. Recent reports indicated that the biological effect of H19 is regulated by steroid hormones. Mammary and intrauterine steroid hormones have been shown to regulate H19 gene expression, with 17-estradiol and progesterone being responsible for upregulation and downregulation, respectively [66]. H19 expression in epithelial and stromal cells is upregulated by chronic hyperprolactinemia, whereas H19 expression is downregulated by dihydrotestosterone. It has been shown that prolactin upregulates H19 via the Janus kinase 2-signal transducer and activator of transcription transduction pathway [67].

## H19 and female congenital infertility

Innate aplasia/hypoplasia of the Müllerian structures of the uterus and the upper portion (2/3) of the vagina in women with normal secondary sexual characteristics and a karyotype of 46, XX are features of the Mayer-Rokitansky-Küster-Hauser (MRKH) syndrome. One of the causes of primary amenorrhea is MRKH. Previous studies identified three cases of Silver-Russell syndrome with MRKH, two of which were linked to substantial hypomethylation of the H19-ICR at 11p15.5 [68]. In a study of 38 patients with Müllerian aplasia, aberrant methylation was observed in 3/16 of the examined areas [69]. These studies suggest a potential link between H19 and female congenital infertility characterized by Müllerian aplasia.

## H19 and unexplained infertility

Studies have revealed a potential association between H19 and unexplained infertility. Korucuoglu et al. discovered that abnormally expressed imprinted IGF2 and H19 genes in their endometrium the endometria of patients with unexplained infertility. The expression of IGF2 mRNA was increased (1.5-fold change, *P* = 0.015), and H19 expression was lower (4-fold change, *P* < 0.0001) in individuals with unexplained infertility [70]. These findings indicate that additional research on imprinted genes is necessary to understand the molecular epigenetic foundations of unexplained infertility.

## Conclusion

This review summarizes the role of lncRNA H19 in infertility and its implication in the mechanisms of infertility from diverse causes, such as PCOS, DOR, endometriosis, UFs, ART-related pathology, and infertility caused by male-related variables, for the first time. There is now enough convincing evidence from various sources to make the case that lncRNA H19 is an important factor impacting infertility.

These findings offer thought-provoking insights into the occurrence and progression of infertility and new molecular targets for prevention and treatment. However, the mechanism by which H19 regulates downstream molecules has not yet been fully explored. Nevertheless, the biological potential of lncRNAs is clear, and the clinical application of H19 and its application in the treatment of reproductive endocrine diseases will be the focus of future research.

## Data Availability

The data used to support the findings of this study are available from the corresponding author upon request.
